# Tessier No. 3 Incomplete Cleft Reconstruction with Alar Transposition and Irregular Z-Plasty

**DOI:** 10.1155/2011/596569

**Published:** 2011-06-21

**Authors:** Orhan Çizmeci, Samet Vasfi Kuvat

**Affiliations:** Department of Plastic and Reconstructive Surgery, İstanbul Medical Faculty, İstanbul University, İstanbul, Turkey

## Abstract

Facial clefts are extremely rare congenital deformities and there are only a few technique reports for surgical reconstruction of clefts in the literature. In this article, we report a Tessier no. 3 incomplete cleft reconstruction with alar transposition and irregular Z-plasty in a 2-year old female patient.

Facial clefts are rare clinical entities observed with an incidence varying between 1.43 and 4.85 per 100,000 births. Other authors have reported an incidence of 9.5 to 34 per 1000 among all cleft cases [1, 2]. The aetiology of the clefts can be explained through a failure in the fusion of the mesoderm during the embryonic process. Still, it is difficult to explain the lateral oro-ocular, some types of nasoocular, and medial oro-ocular clefts with this theory. Some investigators claim that the existence of amniotic bands plays a role in the formation of these clefts [3–5]. 

In 1976, Tessier classified the clefts between 0 and 14 based on the central facial landmarks [6]. The Tessier 3 facial cleft, also called a naso-ocular or a nasomaxillary cleft, results from the disruption of the lateral nasal and maxillary processes. However, in Fearon's surgical classification, this cleft may also be called the orbital cleft because of the orbital involvement [7]. This cleft extends in a direction between the philtrum of the lip and the medial canthus of the eye, involving the nasal ala [3]. 

In a 2-year-old female patient consulted at our clinic due to facial deformity, a Tessier 3 facial incomplete cleft has been detected. The child born as the first child to unrelated healthy parents (mother 23 years, father 29 years of age) through normal vaginal delivery was 51 cm and 3270 gr at birth. In the patient's history, there was no exposure to teratogens or any information pointing towards a genetic syndrome in the families of either parent. For the repair of the deformity in the patient, alar transposition flap (for nasomalar component) and irregular Z-plasty (for lid component) have been planned in an asymmetrical manner considering *the nasomalar and the lid components separately *as suggested by Mishra and Purwar [8] (Figure 1).

The techniques used for the repair of facial clefts are based on the small number of case reports in the literature. These techniques mentioned in the literature for the repair of clefts include the Z-plasty [9], local flaps [1, 10], cheek rotation flap including the lower eyelid [11], rotation and advancement flap of the cheek [2], and the tissue expansion [4, 12] methods. 

In 1990, Resnick and Kawamoto [13] described the interdigitating local flaps technique for the reconstruction of Tesier 4 facial clefts. Longaker et al. [10], on the other hand, have used the superiorly based nasolabial flap for the restoration of two Tessier 4 facial clefts and a multiple cleft case. Giglio et al. [2] claim that these methods are not ideal for the repair of Tessier 3 facial clefts due to excessive scarring. Toth et al. [4] and Menard et al. [12] propose tissue expansion for severe cleft reconstructions; still, this technique may not be necessary for the cases where the repair will be performed using local tissue without an expansion. Giglio et al. [2] have obtained very satisfactory results through the repairs of Tessier 3 facial clefts using rotation and advancement flaps of the cheek. Tessier 4 facial cleft repairs can also be performed using this method, and in fact, this technique described by Van der Meulen [14] was first used for the repair of a Tessier 4 facial cleft. However, the necessity of the rotation and advancement flaps of the cheek technique is disputable for the Tessier 3 clefts that are not severe like in our case, where a satisfactory result, if not perfect, has been obtained through alar transposition flap and irregular Z-plasty. The nasomalar and the lid components of the anomaly were repaired using a alar transposition flap and irregular Z-plasty, respectively. The hyperaemic appearance of the skin on the medial orbita was disturbing, and preoperatively this situation was associated with the similar appearance of the skin in the surrounding area, or in other words, to the thin and low-quality nature of the local skin.

The repair in our patient was tried to be performed through alar transposition flap and irregular Z-plasty, considering the three main components as proposed by Mishra and Purwar [8] (the lip component was intact in our case). These components are (a) the ectropion of the lower eye lid (*Lid component*), (b) the cleft of the upper lip (*Lip component*), (c) and the gap between the nose and the malar area (*Nasomalar component*). Whatever method is chosen, we can confidently claim that these components must be taken into consideration during any repair. 

We are of the opinion that the methods to be applied for the repair of clefts must be investigated in large study groups. Still, a clear treatment algorithm is truly difficult to describe for these rarely observed cases.

## Figures and Tables

**Figure 1:**
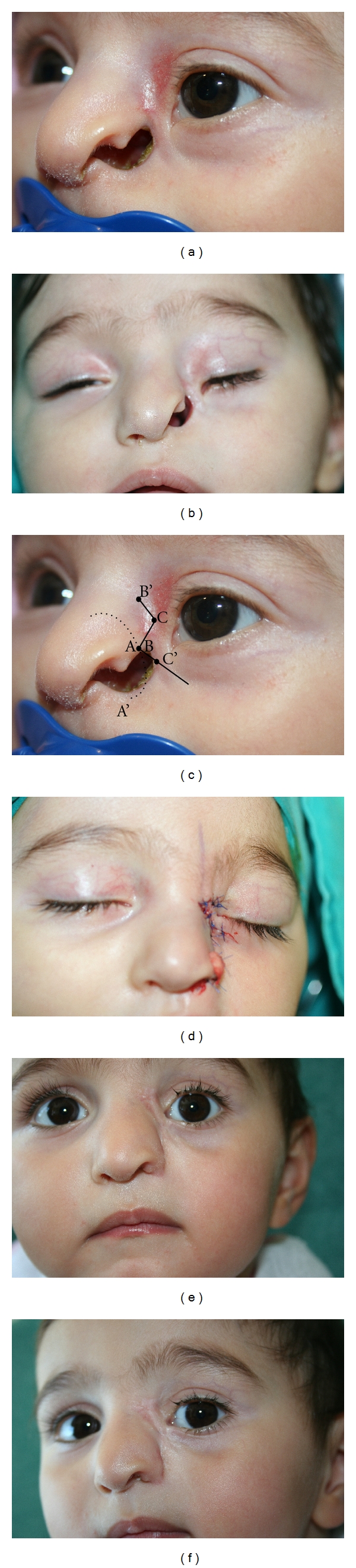
(a) Oblique view of the case at 10-month old. (b) Preoperative frontal view of the case at 2-year old. (c) The dotted parts indicate the incision made for the alar transposition and the area of the flap inset. The straight lines show the incisions for the irregular Z-plasty. (d) Intraoperative photograph after alar transposition flap and irregular Z-plasty. (e)-(f) At 3-month followup.
